# Flies Do Not Jump to Conclusions: Estimation of the Minimum Post-Mortem Interval for a Partly Skeletonized Body Based on Larvae of *Phormia regina* (Diptera: Calliphoridae)

**DOI:** 10.3390/insects12040294

**Published:** 2021-03-28

**Authors:** Senta Niederegger, Gita Mall

**Affiliations:** 1 Department of Forensic Entomology, Institute of Legal Medicine at the University Hospital Jena, 07740 Jena, Germany; 2 Department of Forensic Medicine, Institute of Legal Medicine at the University Hospital Jena, 07740 Jena, Germany; gita.mall@med.uni-jena.de

**Keywords:** thanatology, confession, post-mortem interval, forensic entomology

## Abstract

**Simple Summary:**

Many factors can influence the appearance of human remains. In the case presented here, the remains appeared to be exposed for months, because the bones were visible. Fly maggots collected from the body, however, suggested a much shorter period of only about two weeks. The confession of the perpetrator ultimately confirmed the shorter exposure time of the remains.

**Abstract:**

Skeletonization is often perceived as an indicator of long post-mortem intervals. The finding of feeding larvae of first colonizers, on the other hand, indicates days. We present a case in which both findings were present. Larvae of *Phormia regina*, aged 9 days, and skeletonization of the head and part of the thorax were both found on an unidentified female body. Identification of dentures eventually led to resolution of the case and a confession, which settled the seeming contradiction in favor of forensic entomology.

## 1. Introduction

The fascination with grisly crimes and the resulting supply of crime fiction in books, movies, and shows have increased the popularity of forensic entomology. If, in crime fiction, insects are recognized on a body, they predominantly lead to resolution of the case, sometimes with as little as one maggot. In casework, however, entomological evidence is oftentimes not valued as highly as other indicators. Cases in which entomological estimations are independently verified through confessions can therefore be of valuable help for forensic entomologists [1]. Unfortunately, not many case reports are published [2], and of those, even fewer contain confirmation of the estimated minimal post-mortem intervals (mPMIs) [3,4]. Oftentimes, only witness statements about when the victim was last seen alive are available [5,6].

We present a case in which entomological evidence was unappreciated because it seemingly contradicted another taphonomic indicator that suggested a far longer postmortem interval.

## 2. The Case

**Figure 1:**
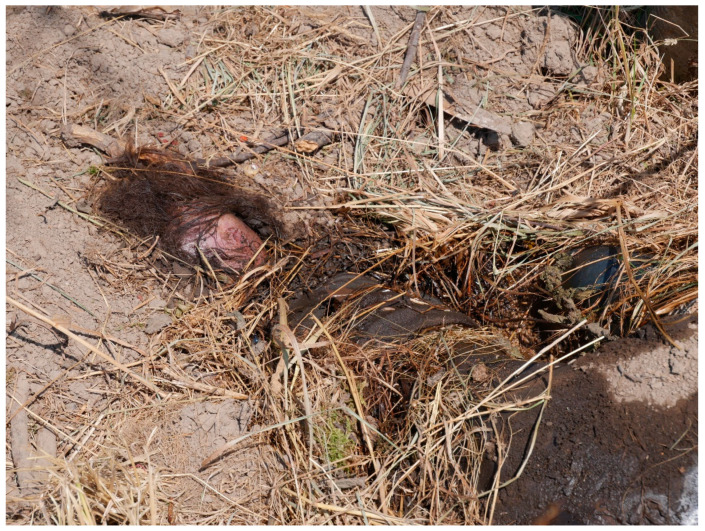
Partly skeletonized human remains found at the edge of a cornfield.

At the beginning of July, in a rural part of Germany, a dog walker discovered the body of an unidentified woman in a pit at the side of a dirty road bordering on a cornfield. The body was deposited on its right side in a fetal position. It was partly covered with soil and partly skeletonized (Figure 1); the skull and a number of ribs were visible. Police personnel collected live third instar larvae at the scene. Photographic evidence showed no presence of puparia.

Due to skeletonization, the doctors examining the body during autopsy the next day estimated the time of death to have been several weeks, if not months, before discovery. The buried parts of the body, however, showed only slight decomposition. Medical examiners also found conspicuous dental work, which resulted in a postmortem pantomogramm (OPG) of the victim’s dentures. No additional entomological evidence was collected during autopsy.

Investigators were not able to identify the woman. No missing person’s report from previous months matched the description. Interrogation of witnesses did not yield any meaningful results. About three weeks after discovery of the body, the case was therefore going to be presented on television to gain information from the public. Investigators requested a forensic entomological expertise before airing the TV show for a more accurate assessment of the possible time of deposition of the body.

### 2.1. Analysis of Insect Evidence

Maggots recovered from the scene reached the Institute of Legal Medicine on the day of their collection. They were divided into three groups: one was frozen, one was transferred to 70% ethanol, and one was stored in the refrigerator. The forensic entomologist was informed about it on the next day after the autopsy, and fortunately, the maggots from the refrigerator were still alive (after about 15 h at +5 °C). The 135 maggots previously stored in 70% ethanol or frozen were hot-water-fixated even after that, and the 50 largest individuals were measured. The average length of the 50 largest larvae was 16.05 mm (size range: 15.30–17.32 mm; median: 16.11 mm; standard deviation: ±0.57 mm). All the larvae collected were identified as *Phormia regina* (Meigen, 1826) (Diptera: Calliphoridae) [7,8]. The living larvae were placed on minced meat and kept at 24 °C in the laboratory. After two days in the lab, the first puparia were formed, and nine days after finding of the body, a large number of adult *P. regina* [9] emerged.

According to temperature data from a nearby weather station (8 km north from the location where the body was found), the average daily temperature for 18 days prior to the finding was 17.7 °C. Based on published data from Nunez-Vazquez et al. [10], *P. regina* requires 12 days at 15 °C and 5.5 days at 20 °C to reach a length of 16 mm. For 17.7 °C, therefore, 9–10 days’ developmental time were assessed.

Accumulated degree-day (ADD) data for the emergence of adult *P. regina* from Nabity et al. [11] are 202 ADD at 15 °C and 205 ADD at 20 °C with “investigator-preferred minimum” [11] and a base temperature of 10 °C (Table 1).

Emergence of adult flies after nine days in the laboratory at 24 °C, therefore, accumulated to 126 ADD. The day of the discovery (day 0) of the body was added with 0 ADD due to 15 h in the refrigerator at 5 °C, which left 76–79 ADD prior to discovery. For each day before discovery, the respective average daily temperature was calculated from weather data (Table 1). The most likely day of egg deposition was determined to be nine days prior to the discovery of the body. Weather data showed no rainfall or cold temperature episode for this or the previous day.

With the age of *P. regina* established to have been 9 days based on both results, we were confident that this was the most likely minimum time that had elapsed since the deposition of the body.

During the TV show, however, the most probable PMI was still given as multiple weeks up to several months on grounds of the skeletonization of the head and the thorax.

### 2.2. Victim Identification

After the TV show, police received multiple tips from viewers. Especially, the OPG and the dentures attracted a lot of attention. Dental assistants pointed out that dental prostheses oftentimes feature serial numbers, and one was indeed detected in the artificial teeth. Investigators were able to find the dentist, and he identified one of his patients. One month after the discovery of the body, the victim was unequivocally identified through her dental prosthesis.

### 2.3. Suspect Identification

After the successful identification, the victim’s apartment was located five weeks after the discovery of her body. Interestingly, the mailbox was empty. This prompted investigators to monitor the building’s main entrance to find out who was in possession of a key to her mailbox. Only two days later, a man was observed emptying the victim’s mailbox and leaving the apartment building. He was soon identified as a resident of a nearby apartment building.

During police questioning, the suspect confessed to killing the victim. He strangled her during the course of an argument over the future of their secret relationship and left the body in the bathroom. Two days later, he decided to discard the remains before suspicion would arise, and bought a large suitcase. Four days after the act, the victim’s body was transported to a remote cornfield, where it was discovered nine days later, partly skeletonized and with larvae of *P. regina*.

## 3. Discussion

For the entomological expertise in this case, publications analyzing US populations of *P. regina* were used. This could be seen as the wrong dataset for European populations. Marchenko [12] worked with Eastern European populations of the species but calculated the pupal stage of *P. regina* to being short. His data did not correspond to our results of 7–8 days for this development period. Other publications were more comparable [11,12,13,14]. Due to its very detailed data, Nabity et al.’s study [11] was exclusively used for our calculations of accumulated degree-days.

A large number of variables affects decomposition and taphonomic processes. The interpretation of post-mortem interval determination can therefore be deceptive [15]. Skeletonization is oftentimes expected to occur only after long exposure times. This, however, might only be true in the absence of or with minimal insect activity. Studies on the decomposition of pig carcasses illustrate this phenomenon. In Matuszewski et al. [16], clothed and unclothed pig carcasses with different body masses were studied. In some cases, the onset of active decay was detected as early as two days after death. Anton et al. [17] had medium-size pig carcasses in summer, going from the fresh stage to the remains stage, with extensive skeletonization within 8 days. In this case, the larvae themselves, due to the diminished accessibility of other body parts, probably accelerated skeletonization.

In the case report presented here, entomological expertise was not valued as evidence and thus mostly disregarded during the investigation. Fortunately, the means to identify the victim were found in the serial number of the dental prosthesis. Would that not have been possible, the case might have remained unsolved. Even if a missing person report would have been filed eventually, investigators could have rejected it due to the presumption of an older age of the remains.

When human remains are found, it is therefore important to evaluate all processes and give them equal prominence in estimating plausible post-mortem intervals.

## 4. Conclusions

Insect evidence resulted in an estimation of time since the deposition of the body of less than two weeks and was regarded as potentially erroneous in light of the skeletonization. The time frame established by forensic entomology was later confirmed by the confession. Such results strengthen the scientific value of entomological expertise in casework and its standing in criminal investigations.

## Figures and Tables

**Table 1:** Calculation of accumulated degree-days (ADD) for *Phormia regina*, based on temperature information from the nearest weather station. bold: estimated day of body deposition

Day	Daily Average °C	Threshold °C	Degree-Days	ADD
−10	15.5	10	5.5	209.9
**−** **9**	**17.4**	**10**	**7.4**	**204.4**
−8	17.3	10	7.3	197.0
−7	19.2	10	9.2	189.7
−6	22.5	10	12.5	180.5
−5	17.2	10	7.2	168.0
−4	15.8	10	5.8	160.8
−3	16.7	10	6.7	155.0
−2	19.4	10	9.4	148.3
−1	22.9	10	12.9	138.9
Day 0	5.0	10	0	126.0
Lab1–9	24.0	10	14	126.0

## Data Availability

The data presented in this study are available on request from the corresponding author.
